# CD133^+^ circulating haematopoietic progenitor cells predict for response to sorafenib plus erlotinib in non-small cell lung cancer patients

**DOI:** 10.1038/sj.bjc.6605477

**Published:** 2009-12-15

**Authors:** L Vroling, J S W Lind, R R de Haas, H M W Verheul, V W M van Hinsbergh, H J Broxterman, E F Smit

**Affiliations:** 1 Department of Medical Oncology, VU University Medical Center Amsterdam, De Boelelaan 1117, Amsterdam 1081 HV, The Netherlands; 2 Department of Pulmonary Diseases, VU University Medical Center Amsterdam, De Boelelaan 1117, Amsterdam 1081 HV, The Netherlands; 3 Department of Physiology, VU University Medical Center Amsterdam, De Boelelaan 1117, Amsterdam 1081 HV, The Netherlands

**Keywords:** CECs, HPCs, biomarker, angiogenesis inhibitors, sorafenib, bevacizumab, erlotinib

## Abstract

**Background:**

Blood-based biomarkers may be particularly useful for patient selection and prediction of treatment response for angiogenesis inhibitors. Circulating endothelial cells (CECs) and haematopoietic progenitor cells (HPCs) might have a role in tumour angiogenesis and in tumour growth. Measurement of CECs and HPCs in the blood of patients could be a simple, non-invasive way to monitor or predict responses to treatment.

**Methods:**

(VEGFR2^+^) CECs_,_ (CD133^+^) HPCs, plasma vascular endothelial growth factor (VEGF) and erythropoietin were measured in blood from 25 non-small cell lung cancer (NSCLC) patients before and during treatment with sorafenib plus erlotinib (SO/ER). In order to assess the drug specificity of changes in CECs and HPCs, 18 patients treated with bevacizumab plus erlotinib (BV/ER) and 10 patients with erlotinib (ER) monotherapy were studied. Response was measured in all patient groups by Response Evaluation Criteria in Solid Tumors (RECIST).

**Results:**

At day 7, SO/ER-treated patients showed a three-fold increase in CECs (*P*<0.0001) comparable to BV/ER-treated patients (*P*<0.01), and the CECs did not change with erlotinib treatment (*P*=0.8). At day 7, CD133^+^/HPCs decreased with SO/ER treatment (*P*<0.0001). HPC numbers did not change with either BV/ER or erlotinib. In SO/ER-treated patients pre-treatment CD133^+^/HPCs were significantly lower in responders (*P*=0.01) and pre-treatment CD133^+^/HPC numbers lower than the median correlated with a longer time-to-progression (TTP) (*P*=0.037).

**Conclusion:**

Pre-treatment CD133^+^/HPCs are a promising candidate biomarker to further explore for use in selecting NSCLC patients who might benefit from SO/ER treatment.

Anti-angiogenic agents used to target tumour neovascularization can be classified into two groups: agents binding the vascular endothelial growth factor (VEGF), such as the humanised monoclonal antibody (Mab) bevacizumab or the soluble decoy receptor VEGF Trap and second, the receptor tyrosine kinase inhibitors (TKIs) targeting the VEGF receptor family, such as sunitinib and sorafenib.

Bevacizumab has proven efficacy against several solid tumours (Presta *et al*, 1997), amongst others against non-small cell lung cancer (NSCLC). A phase III study by Sandler *et al* (2006) showed a survival benefit in advanced stage NSCLC patients when bevacizumab was combined with chemotherapy in first-line setting. This was recently confirmed by Manegold *et al* (2008). Moreover, a phase I/II trial combining the epidermal growth factor receptor (EGFR) inhibitor erlotinib or chemotherapy with bevacizumab resulted in higher response rates and longer median progression-free survival (PFS) in both bevacizumab containing arms (Herbst *et al*, 2007).

The orally available anti-angiogenic TKI, sorafenib is a multi-targeted inhibitor of raf-kinase, VEGFR1, VEGFR2, VEGFR3, PDGFR-*β*, Flt3 and c-kit, which has been approved in the treatment of both advanced renal cell carcinoma and hepatocelullar carcinoma. There is an emerging evidence of clinical anti-tumour activity in NSCLC (Gatzemeier *et al*, 2006; Schiller *et al*, 2008).

Although anti-tumour activity is achieved by these anti-angiogenic drugs either as monotherapy or in combination with chemotherapy or with other targeted agents, it remains unclear which patient will benefit most from treatment (Broxterman *et al*, 2009). Biomarkers that potentially predict treatment response, in order to guide patient selection and/or monitor early response, are currently being investigated (Le Tourneau *et al*, 2008; Pathak *et al*, 2008).

Particularly attractive biomarkers might be circulating endothelial cells (CECs), as these may reflect damage to the tumour vasculature or circulating haematopoietic progenitor cells (HPCs), as they have been proposed to reflect a pro-angiogenic cell population, which may respond to increased hypoxia by effective anti-angiogenic therapy (Jain and Duda, 2003; Shaked *et al*, 2006; Zurita *et al*, 2009).

No studies are currently available assessing the effects of anti-angiogenic agents in NSCLC patients on CECs and HPCs. In this study we evaluated (1) changes in CECs, defined as CD34^bright^/CD45^−^/(VEGFR2^+^), and HPCs, defined as CD34^bright^/CD45^dim^/(CD133^+^), in NSCLC patients treated with anti-angiogenic therapy with sorafenib plus erlotinib (SO/ER); (2) we investigated the specificity of these changes by comparing these with a control group of patients receiving bevacizumab plus erlotinib (BV/ER) or monotherapy erlotinib, and (3) we correlated the changes of the biomarkers with treatment response in the SO/ER patient group.

## Materials and methods

### Patient and study design

From November 2007 to October 2008 25 chemotherapy-naive patients with advanced NSCLC who were treated at the VU University Medical Center in a phase II trial combining erlotinib (150 mg day^−1^) and sorafenib (400 mg twice daily) were enrolled in this study (Lind *et al*, 2009a).

For comparison, a cohort of 18 NSCLC patients treated with bevacizumab (15 mg kg^−1^ intravenously every 21 days) plus erlotinib (150 mg day^−1^ orally) in a phase II trial (Groen *et al*, 2007) and a control group of 10 patients treated with whole brain radiotherapy (WBRT) plus concurrent erlotinib for brain metastases from NSCLC in phase I trial were evaluated (Lind *et al*, 2009b). These control group patients received erlotinib 1 week before and during WBRT (30 Gy in 10 once-daily fractions) and continued maintenance erlotinib (150 mg day^−1^) until unacceptable toxicity or disease progression.

Before study entry, all patients provided written informed consent in accordance with the national and institutional guidelines, which strictly adhere to the principles of the Declaration of Helsinki and ICH/Good Clinical Practice.

Peripheral blood (PB) was taken from all patients on three occasions: before start of treatment, day (D) 0, and 7 and 21 days after starting drug treatment, D7 and D21, respectively. Computed tomography was carried out before and 6 weeks after the start of treatment to assess clinical response according to the Response Evaluation Criteria in Solid Tumors (RECIST) (Therasse *et al*, 2000). In the SO/ER-treated patients response incorporating primary tumour cavitations, as proposed by Crabb *et al* (2009), were also determined. Clinical responses after 6 weeks of treatment were used to examine a possible relation with VEGF and erythropoietin (EPO, in SO/ER-treated patients only) levels and the cellular biomarkers.

### Evaluation of cells and plasma biomarkers

Blood from SO/ER-treated patients was collected in EDTA tubes and the circulating HPCs and CECs were measured using a full-blood flow cytometric method as previously described (Vroling *et al*, 2007, 2009). (CD133^+^) HPCs were defined as CD34^bright^/CD45^dim^ (and CD133^+^) and CECs were defined as CD34^bright^/CD45^neg^ and largely positive for VEGFR2. The antibodies used to carry out analysis in this study included CD45–FITC, CD34–APC, CD133–PE, VEGFR2–PE and viability marker 7-AAD. Cell populations were measured by flow cytometry (FACSCalibur, BD Bioscience, Breda, The Netherlands) in a total volume of 1.5 ml full blood and appropriate IgG isotypes were used as a control. Circulating cells from the comparison groups (monotherapy erlotinib and BV/ER) were assessed with CD45–FITC, CD34–APC and 7-AAD. Cell populations of the total white blood cells (WBC) were calculated as number ml^−1^ of blood using blood cell count measured on a Sysmex. EDTA blood was used for the collection of plasma, which was stored at −80°C until further analysis. Plasma VEGF and EPO levels were measured in duplicate with the enzyme-linked immunosorbent assay (ELISA) kit (R&D Systems, Minneapolis, MN, USA).

### Statistical analyses

Circulating cell populations (numbers ml^−1^), plasma levels of VEGF (pg ml^−1^) and EPO (mIU ml^−1^) were expressed as median (range). Continuous variables between groups were compared with Mann–Whitney *U*-test. To compare the blood-based parameters at pre-treatment with those during treatment (D0, D7 and D21) the Wilcoxon's signed-rank test was used. Time-to-progression (TTP) was determined as the time period from start of treatment to the time of documented disease progression with a minimum follow-up period of 6 months. Overall survival (OS) was the time between the first day of treatment and the date of death or the date on which patients were last known to be alive. Correlations of the circulating cells with TTP and overall OS were determined with the Kaplan–Meier method and its associated log-rank statistic. Responders were defined as those achieving partial response (PR) according to RECIST and non-responders as those with stable disease (SD) or progressive disease (PD) after 6 weeks of treatment. Differences were considered statistically significant when *P*<0.05 (two-tailed). As this was an exploratory study, no correction for multiple testing was done. Statistical analyses were carried out with SPSS (SPSS for Windows 15.0, SPSS, Chicago, IL, USA) software programme.

## Results

### Patient characteristics and treatment response

In total, 53 NSCLC patients in three study groups were investigated. Patients treated with SO/ER included in this study (14 males and 11 females) had a median age of 60 years (range 41–78) at the start of treatment. The clinical response of patients was assessed using RECIST criteria (Therasse *et al*, 2000). Thirteen out of the 25 SO/ER-treated patients had developed cavitations in the primary tumour at week 6 of evaluation. For these patients, responses incorporating cavitations were also correlated with the circulating cells and plasma parameters. Patients treated with SO/ER had a median TTP of 6 months (range 0.82–12.7) and an OS of 12.4 months (range 2.3–16.0). Patients treated with BV/ER had a median TTP of 6.8 months (range 3.2–10.3) and an OS of 6.9 months (range 5.4–8.3). Further, patient and tumour characteristics are summarised in Table 1.

### Definitions of HPCs and CECs populations

**Figure 1:**
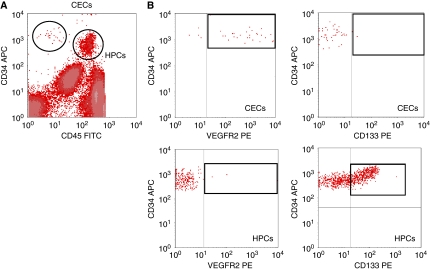
Full blood analysis of circulating endothelial cells (CECs) and haematopoietic progenitor cells (HPCs) in a non-small cell lung cancer (NSCLC) patient. (**A**) Detection of total CECs and HPCs based on marker expression of CD45 and CD34. CECs by CD45^neg^/CD34^bright^ and HPCs defined by CD45^dim^/CD34^bright^. (**B**) Expression of VEGFR2 and CD133 on CECs and HPCs as indicated by the boxes.

The FACS analysis of a blood sample of a representative patient from the study is shown in Figure 1. As described previously for renal cell cancer patients (Vroling *et al*, 2009), two distinct CD34^+^ cell populations, which differ in the expression of the leucocyte marker CD45 and progenitor marker CD133, were identified in NSCLC patients. HPCs are the CD45^dim^/CD34^bright^ cells, which are largely CD133^pos^. The cutoff value for CD133 positivity in HPCs was determined based on isotype control and was adjusted for every individual sample. CECs are CD34^bright^, which are almost uniformly positive for VEGFR2, but lack both markers CD45 and CD133 as previously defined (Vroling *et al*, 2007, 2009) (Figure 1).

### Marked decrease of HPCs during treatment with TKI sorafenib

**Figure 2:**
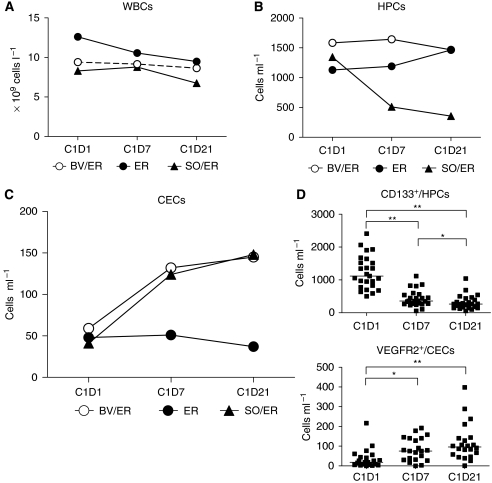
Blood cell populations during anti-angiogenic treatment. (**A**) The total white blood cell (WBC) levels show little change during treatment with sorafenib plus erlotinib (SO/ER) and bevacizumab plus erlotinib (BV/ER). (**B**) Although BV/ER and ER treatment do not show a decrease in viable haematopoietic progenitor cell (HPC) numbers, treatment with tyrosine kinase inhibitor (TKI) SO/ER shows a significant marked depression of the viable HPCs after D7 (*P*<0.001) and D21 (*P*<0.001). (**C**) TKI SO and monoclonal antibody (Mab) BV combined with ER lead to an increase in viable circulating endothelial cells (CECs) after D7 (*P*<0.01 and *P*<0.01, respectively) and further increased after D21 (*P*<0.001 and *P*<0.01, respectively), which was not observed with monotherapy ER. (**D**) The viable CD133^+^/HPCs and VEGFR2^+^/CECs sub-populations measured in SO/ER patients showed a respective decrease and increase in parallel to the total population of HPCs and CECs. ^*^P<0.05, ^**^P<0.001.

The total WBC count showed little change during treatment with SO/ER, BV/ER or monotherapy erlotinib (Figure 2A). Although no leucopenia was observed during any of the treatment regimens, we found a marked decrease of HPCs (Figure 2B) after 7 days of treatment with SO/ER (median from 1345 cells ml^−1^ at D0 to 511 cells ml^−1^ on D7, *P*<0.001), which decreased further after 3 weeks of treatment (to a median of 356 cells ml^−1^, *P*<0.0001). The sub-population of CD133^+^/HPCs also showed a similar decrease (from 1086 cells ml^−1^ at D0 to 361 cells ml^−1^ on D7 (*P*<0.0001) to 264 cells ml^−1^ on D21 (*P*<0.0001)). In the patient groups, treated with either BV/ER or monotherapy erlotinib, the HPC numbers did not change significantly (Figure 2B).

### Anti-angiogenic treatment-specific increase of CECs

**Figure 3:**
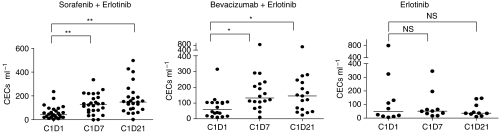
Specificity of increase in circulating endothelial cells (CECs). Anti-angiogenic treatment with direct VEGFR tyrosine kinase inhibitor (TKI) sorafenib, as well as indirect vascular endothelial growth factor (VEGF) withdrawal by monoclonal antibody (Mab) bevacizumab in combination with anti-epidermal growth factor receptor (EGFR) inhibitor erlotinib showed an increase in CECs after 7 days and 21 days of treatment. No change in CEC numbers was observed in control patients treated with TKI erlotinib. ^*^P<0.01, ^**^<0.001.

Patients treated with SO/ER showed an increase in the number of CECs, which was also found in patients treated with BV/ER but not with monotherapy erlotinib (Figure 2C). SO/ER-treated patients showed a three-fold increase in CECs on D7 (median, from 41 to 124 cells ml^−1^, *P*=0.001) and BV/ER-treated patients more than a two-fold increase in CECs (median, from 59 to 132 cells ml^−1^, *P*=0.002). CECs during monotherapy erlotinib did not change after 7 days of treatment (median, from 48 to 51 cells ml^−1^, *P*=0.8). The VEGFR2 positive fraction of CECs enumerated in SO/ER-treated patients followed the same trend as total CECs (Figure 2D). In both patient groups treated with either a TKI or anti-VEGF, Mab-angiogenesis inhibitor CECs further increased after 3 weeks of treatment in contrast to patients treated with monotherapy erlotinib (Figure 3).

### Response and biomarkers

**Figure 4:**
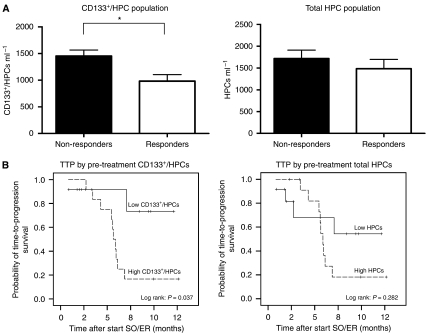
Boxplots and Kaplan–Meier curves, according to peripheral blood circulating CD133^+^/haematopoietic progenitor cells (HPCs) in sorafenib plus erlotinib (SO/ER)-treated non-small cell lung cancer (NSCLC) patients. (**A**) Mean pre-treatment numbers of CD133^+^/HPC of non-responders (*n*=11) and responders (*n*=13). Responses are according to Response Evaluation Criteria in Solid Tumors (RECIST) incorporated cavitations. ^*^=*P*<0.05. (**B**) Time-to-progression (TTP) according to RECIST correlated with pre-treatment CD133^+^/HPCs and the total population of HPCs with a median cutoff value of 1086 and 1345 cells ml^−1^, respectively. ‘Low’ indicates (CD133^+^)/HPCs at baseline below the median and ‘high’ indicates (CD133^+^)/HPCs at baseline higher than the median.

In order to analyse the possible relations of biomarker values with response we used RECIST criteria and RECIST criteria taking into account tumour cavitations as recently suggested by Crabb *et al* (2009). CD133^+^/HPCs were not significantly correlated with the response when RECIST was not adjusted for tumour cavitations. When the response was corrected for cavitations, pre-treatment levels of CD133^+^/HPCs but not the total HPCs, were significantly lower in responding (PR) patients compared with non-responding (SD+PD) patients treated with SO/ER (*P*=0.01 and *P*=0.28 respectively, Figure 4A). Nine out of 12 patients with lower than the median of 1086 CD133^+^/HPCs ml^−1^ and 4 out 12 with higher than the median of CD133^+^/HPCs showed a response (PR). Patients with low CD133^+^/HPCs (lower than the median of 1086 cells ml^−1^) had a significantly longer TTP than those with CD133^+^/HPCs higher than the median pre-treatment levels, which was again not seen for the total population of HPCs (*P*<0.05 and *P*=0.334 respectively, Figure 4B). TTP was calculated from RECIST not corrected for tumour cavitations, as this is currently not validated. At the time of analyses CD133^+^/HPCs did not correlate with OS. Pre-treatment CECs or changes in CECs after 7 or 21 days in patients treated with SO/ER did not correlate with TTP, OS or response (according RECIST) with or without adjustments for cavitations. The latter was comparable for patients treated with BV/ER.

### Plasma VEGF and EPO levels in NSCLC patients

**Figure 5:**
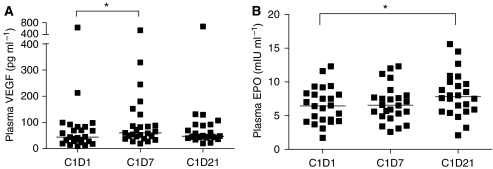
Plasma vascular endothelial growth factor (VEGF) and erythropoietin (EPO) levels in non-small cell lung cancer (NSCLC) patients treated with SO/ER. (**A**) Free plasma VEGF levels (pg ml^−1^) did not change significantly during 21 days of sorafenib plus erlotinib (SO/ER) (*n*=25). ^*^P<0.01. (**B**) Plasma EPO levels (mIU ml^−1^) in NSCLC patients showed a slight increase during sorafenib treatment. (D21, ^*^P<0.01).

Patients treated with SO/ER showed a slight non-significant increase in plasma VEGF during 21 days of treatment (Figure 5A), which was similar in patients treated with monotherapy erlotinib (data not shown). BV/ER-treated patients showed a significant decrease in plasma VEGF levels after 7 days (median decreased from 134 to 25 pg ml^−1^, *P*=0.002, data not shown), probably because the used ELISA-kit measures only free VEGF (Loupakis *et al*, 2007). No correlation was found between pre-treatment plasma VEGF levels and treatment outcome. Moreover, we found no significant relationship between the (VEGFR2^+^) CECs, (CD133^+^) HPCs and pre-treatment plasma VEGF in any patient group (data not shown).

To investigate if plasma EPO levels can serve as a surrogate pharmacodynamic biomarker or may have a possible relation with CECs and HPCs the plasma EPO levels were determined in the SO/ER-treated patients. Minor changes in plasma EPO were observed (median, from 6.5 on D0 to 6.6 on D7 to 7.8 mIU ml^−1^ on D21, Figure 5B) which were all in the normal range of EPO levels (3.1–14.9 mIU ml^−1^). Pre-treatment plasma EPO levels showed no correlation with circulating cell numbers or patient response (data not shown).

## Discussion

We monitored, to our knowledge for the first time, VEGFR2-positive circulating endothelial cells and CD133-positive haematopoietic progenitor cells in NSCLC patients treated with the anti-angiogenic agent SO combined with ER. The second objective of the study was to provide data on the specificity of the measured changes in CECs and HPCs for SO/ER treatment. For this purpose data from two smaller groups of patients were obtained. The most important findings we report here are:

1. VEGFR2^+^ CECs increase in an anti-VEGF treatment-specific manner, with a similar two- to three-fold increase (on D7 or D21) after treatment with the VEGFR–TKI sorafenib or anti-VEGF antibody bevacizumab, both combined with the EGFR–TKI erlotinib, but not with erlotinib monotherapy (see Figure 2C).
2. CD133^+^/HPCs markedly decreased with >60% on D7 and about 75% on D21 of treatment with SO/ER. This effect is not seen during treatment with BV/ER or monotherapy erlotinib with whole brain radiotherapy.
3. The pre-treatment CD133^+^/HPC numbers predicted for response incorporating tumour cavitations to SO/ER therapy. In addition, TTP defined by RECIST was significantly longer in the patient group with a lower than median number of CD133^+^ HPCs.

Circulating endothelial cells are a very rare cell population, normally defined as mature endothelial cells shed from the vasculature and are considered to be a useful biomarker of vascular damage (Willett *et al*, 2004; Duda *et al*, 2006; Batchelor *et al*, 2007; Norden-Zfoni *et al*, 2007). In addition, the possibility to monitor circulating endothelial progenitor cells (CEPs) has been proposed as an attractive biomarker for anti-angiogenic agents after the seminal identification of circulating progenitors, which could grow out to highly proliferative endothelial outgrowth cells (EOCs) (Ingram *et al*, 2005; Shaked *et al*, 2006). Although originally CEPs were discriminated from mature CECs mainly by the presence or absence of CD133 progenitor markers, more recent data have cast serious doubts on this premise (Timmermans *et al*, 2008; Yoder and Ingram, 2009).

The population of CECs that we have monitored has a phenotype consistent with a population containing the precursor for EOCs, namely CD34^+^, VEGFR2^+^, but CD45^−^ and CD133^−^ (see Figure 1) according to Case *et al* (2007) and Timmermans *et al* (2007). However, whether CECs are a mixed mature/progenitor population will only be determined once these very rare cells can be sorted after a unique specific marker of the endothelial progenitor cell will have been identified (Yoder and Ingram, 2009).

In this study, the CEC population did not predict for response to SO/ER or BV/ER therapy. Regarding the explanations for the lack of correlation between pre-treatment values or increases in CECs and response one can only speculate. Our finding of an increase in CECs in SO/ER or BV/ER, but not monotherapy erlotinib-treated patients is consistent with our earlier finding of a similar increase in renal cell cancer patients treated with the VEGFR–TKI sunitinib (Vroling *et al*, 2009). Therefore, the increase of this cell population is likely to be a pharmacodynamic marker for VEGF/VEGFR signalling-inhibitor therapy.

Preclinical and early clinical data (phase I and phase II trials) have supported combined inhibition of VEGFR and EGFR pathways in NSCLC (Pennell and Lynch, 2009). Activating EGFR mutations are predictive of response to EGFR inhibitors with reported response rates of upto 75%. In the specific group studied here, six out of eight (75%) patients with an activating EGFR mutation showed a response (PR). Of the WT EGFR group 6 out of 13 (46%) patients showed a response (PR). This is higher than the rates reported for erlotinib monotherapy and implies a significant contribution of sorafenib on the clinical outcome with this combination treatment. The currently identified CD133^+^ progenitor cell population might be of help in future evaluation of this type of combination therapy.

Unlike CECs, the total HPC population showed a marked decrease during SO/ER treatment in contrast to patients treated with BV/ER or monotherapy erlotinib with WBRT. Such a decrease of the HPCs also occurs in RCC patients treated with sunitinib (Vroling *et al*, 2009) and might be related to the Flt-3 inhibitory effects of both TKI sorafenib and sunitinib, as Flt3-signalling is required for HPC proliferation and mobilisation into the circulation (Gabbianelli *et al*, 1995; Faivre *et al*, 2007; Kumar *et al*, 2009). In this study, the pre-treatment level of the CD133^+^/HPCs was significantly higher in the non-responders to SO/ER therapy, and the group with higher than median CD133^+^/HPC values showed a shorter TTP. Also, the decrease in CD133^+^/HPCs on D7 was much smaller in patients with longer TTP (data not shown), which may be a reflection of the lower HPC mobilisation from the bone marrow into the circulation in those patients. Although the inhibition of circulating HPC numbers is very likely a pharmacological effect of sorafenib, the correlation with the anti-tumour effect (of the sorafenib plus erlotinib combination) may or may not be indicative of a role of this population in tumour growth. In contrast to the hypothesised effects of populations of circulating endothelial progenitors, a pro-angiogenic role of HPCs and CD133^+^ sub-populations of HPCs recruited from the bone marrow to hypoxic or tumour tissues (Kerbel, 2008; Burt *et al*, 2009) is consistently reported. Thus, there is wide agreement on the positive role of CD133^+^/CD34^bright^/CD45^dim^ HPCs in (tumour) adult neovascularization. The ongoing debate is on the evidence for the existence in the adult of bone marrow-derived progenitors for potent long-term endothelial layer forming cells in addition to the progenitors for cell populations that promote tumour neovascularization indirectly by secreting growth factors. However, present studies point to a limited incorporation of bone marrow-derived cells into the endothelium of tumours (Peters *et al*, 2005). Many recent studies and reviews discuss the properties of pro-angiogenic cell populations or ‘circulating angiogenic cells’ in depth (Case *et al*, 2007; Timmermans *et al*, 2007, 2008; Hirschi *et al*, 2008; Gao *et al*, 2009; Yoder and Ingram, 2009).

A specific role of CD133^+^/HPCs has been suggested in pulmonary vascular remodelling in patients with obstructive pulmonary disease (Asosingh *et al*, 2008) or idiopathic pulmonary arterial hypertension (Diez *et al*, 2007). Thus, it seems plausible that the production and release of cytokines, chemokines and proteolytic enzymes (VEGF, SDF-1 and metalloproteases) by the lung tumour tissue will increase the mobilisation of CD133^+^/HPCs from the bone marrow, which may be home to hypoxic tumour tissue and have a fundamental role in tumour neovascularization and progression (Kerbel, 2008; Shojaei and Ferrara, 2008).

In this scenario, part of the anti-tumour effect of sorafenib, but also sunitinib, may be related to the suppression of mobilisation of (CD133^+^) HPCs from the bone marrow. Other studies also suggest that high numbers of circulating CD133^+^/HPCs are related to a worse outcome in cancer patients. CD133 mRNA from PB cells, which is predominantly derived from the HPCs was shown to be an independent prognostic factor for patient survival (Mehra *et al*, 2006).

A very recent study showed that higher pre-treatment levels of CD133^+^/CD34^+^/CD45^dim^ cells (called circulating progenitor cells in that study) were associated with the worst outcome of suntinib therapy in hepatocellular carcinoma (Zhu *et al*, 2009).

Altogether, these results fit with the concept of the pro-angiogenic capacity of HPCs in tumour neovascularization and might indicate a causative role of the suppression of these cells by the anti-angiogenic TKIs sorafenib and/or sunitinb to their anti-tumour effects and patient responses. As this cell population is well defined, it is also amenable to analysis at the RNA level in PB mononuclear cells.

In addition, plasma levels of VEGF and EPO (representing a larger repertoire of cytokines/growth factors) are known to be increased in mice or patients upon treatment with anti-VEGF therapy (Ebos *et al*, 2007; Vroling *et al*, 2009) and have early on been considered as candidate biomarkers for the effectivity of this type of therapy. In this study, VEGF and EPO, were not predictive of response or TTP to SO/ER-combination therapy. Dowlati *et al* (2008) found that VEGF was predictive for response in NSCLC patients treated with bevacizumab, but no relation was found with survival.

An important difficulty in defining or identifying biomarkers is the evaluation of the endpoint of the actual responses of the patients. RECIST is the most widely used method for assessing the responses of patients (Therasse *et al*, 2000), but has been critically updated by Karrison *et al* (2007) and Verweij *et al* (2009). In addition Crabb *et al* (2009) suggest that response assessment might be improved in NSCLC patients, treated with angiogenesis inhibitors, by incorporating cavitation into volume assessment for target lesions potentially altering treatment outcomes. In that study, marked pulmonary cavitation occurred in 24% of patients treated with the angiogenesis inhibitor cediranib plus chemotherapy, which was not seen with chemotherapy alone. Our sorafenib data suggest that correcting for cavitations may be of importance in evaluating potential biomarkers in relation to response. In SO/ER-treated patients 13 out of 25 patients had cavitations because of the treatment. Incorporating these cavitations in response assessment altered the RECIST responses. Pre-treatment numbers of CD133^+^/HPCs were only prognostic for the response if corrected for cavitations.

In our study, several cell populations and plasma markers were evaluated to serve as a potential biomarker during anti-angiogenesis treatment. This introduces the potential problem of multiple testing, which increases the risk to find false-positive relations. Clearly, our study was designed to explore associations that should be confirmed in an independent group of patients.

In conclusion, CECs increased in NSCLC patients treated with SO/ER and BV/ER, but not with erlotinib monotherapy. Thus, this effect can be ascribed to the anti-angiogenic agents. The CD133^+^/HPCs decreased significantly in all patients treated with SO/ER and pre-treatment numbers were significantly lower in responding patients and pre-treatment CD133^+^/HPC numbers correlated with the TTP. CD133^+^/HPCs may therefore be considered for further investigations as a biomarker for selecting patients who are likely to benefit from SO/ER.

## Figures and Tables

**Table 1:** Patient characteristics and response to treatment at 6 weeks

**Characteristic**	**SO/ER**	**BV/ER**	**ER**
	***N* (%)**	***N* (%)**	***N* (%)**
Total	25	18	10
			
*Sex*			
Male	14 (44)	12 (67)	4 (40)
Female	11 (56)	6 (33)	6 (60)
Median age (years) (range)	60 (41–78)	63 (34–80)	68 (59–77)
			
*Smoking status*			
Current or former smoker	20 (80)	18 (100)	9 (90)
Non-smoker	5 (20)	0	1 (10)
			
*ECOG performance status*			
0	17 (68)	5 (28)	2 (20)
1	8 (32)	9 (50)	7 (70)
2	0	4 (22)	1 (10)
			
*Tumour histology*			
Adenocarcinoma	18 (72)	12 (67)	5 (50)
Large cell	2 (8)	5 (28)	4 (40)
Squamous cell	1 (4)	0	1 (10)
BAC	2 (8)	1 (6)	0
NSCLC NOS	2 (8)	0	0
			
*Tumour stage*			
IIIB	7 (28)	5 (28)	1 (10)
IV	18 (72)	13 (72)	9 (90)
			
*Previous treatment*			
Yes	2 (8)	0	5 (50)
No	23 (92)	18 (100)	5 (50)
			
*Response at 6 weeks*			
Partial response	10 (40)	4 (22)	0
Stable disease	12 (48)	10 (56)	8 (80)
Progressive disease	3 (12)^a^	4 (22)	2 (20)
			
*Response at 6 weeks corrected for cavitations*		—	—
Partial response	13 (52)		
Stable disease	9 (36)		
Progressive disease	2 (8)^a^		
			
Median time to progression, months (95% CI)	6.0 (11.7–13.2)	6.8 (3.2–10.3)	6.6 (2.6–10.7)
Median survival, months (95% CI)	12.5 (11.7–13.2)	6.9 (5.4–8.3)	6.0 (0–12.8)

Abbreviations: BAC=bronchioloalveolar carcinoma; BV=bevacizumab; ECOG=Eastern Cooperative Oncology Group; ER=erlotinib; SO=sorafenib.

a One PD patient had no formal evaluation for treatment response because of early off study due to treatment-related toxicity.

## References

[bib1] Asosingh K, Aldred MA, Vasanji A, Drazba J, Sharp J, Farver C, Comhair SA, Xu W, Licina L, Huang L, nand-Apte B, Yoder MC, Tuder RM, Erzurum SC (2008) Circulating angiogenic precursors in idiopathic pulmonary arterial hypertension. Am J Pathol 172(3): 615–627 18258847 10.2353/ajpath.2008.070705 PMC2258264

[bib2] Batchelor TT, Sorensen AG, di Tomaso E, Zhang WT, Duda DG, Cohen KS, Kozak KR, Cahill DP, Chen PJ, Zhu M, Ancukiewicz M, Mrugala MM, Plotkin S, Drappatz J, Louis DN, Ivy P, Scadden DT, Benner T, Loeffler JS, Wen PY, Jain RK (2007) AZD2171, a pan-VEGF receptor tyrosine kinase inhibitor, normalizes tumor vasculature and alleviates edema in glioblastoma patients. Cancer Cell 11(1): 83–95 17222792 10.1016/j.ccr.2006.11.021 PMC2748664

[bib3] Broxterman HJ, Gotink KJ, Verheul HM (2009) Understanding the causes of multidrug resistance in cancer: a comparison of doxorubicin and sunitinib. Drug Resist Updat 12(4–5): 114–126 19648052 10.1016/j.drup.2009.07.001

[bib4] Burt RK, Testori A, Oyama Y, Rodriguez HE, Yaung K, Villa M, Bucha JM, Milanetti F, Sheehan J, Rajamannan N, Pearce WH (2009) Autologous peripheral blood CD133+ cell implantation for limb salvage in patients with critical limb ischemia. Bone Marrow Transplant, e-pub ahead of print. doi: 10.1038/bmt.2009.102 10.1038/bmt.2009.102 PMC3951860 19448678

[bib5] Case J, Mead LE, Bessler WK, Prater D, White HA, Saadatzadeh MR, Bhavsar JR, Yoder MC, Haneline LS, Ingram DA (2007) Human CD34(+)AC133(+)VEGFR-2(+) cells are not endothelial progenitor cells but distinct, primitive hematopoietic progenitors. Exp Hematol 35(7): 1109–1118 17588480 10.1016/j.exphem.2007.04.002

[bib6] Crabb SJ, Patsios D, Sauerbrei E, Ellis PM, Arnold A, Goss G, Leighl NB, Shepherd FA, Powers J, Seymour L, Laurie SA (2009) Tumor cavitation: impact on objective response evaluation in trials of angiogenesis inhibitors in non-small-cell lung cancer. J Clin Oncol 27(3): 404–410 19047292 10.1200/JCO.2008.16.2545

[bib7] Diez M, Barbera JA, Ferrer E, Fernandez-Lloris R, Pizarro S, Roca J, Peinado VI (2007) Plasticity of CD133+ cells: role in pulmonary vascular remodeling. Cardiovasc Res 76(3): 517–527 17850777 10.1016/j.cardiores.2007.08.007

[bib8] Dowlati A, Gray R, Sandler AB, Schiller JH, Johnson DH (2008) Cell adhesion molecules, vascular endothelial growth factor, and basic fibroblast growth factor in patients with non-small cell lung cancer treated with chemotherapy with or without bevacizumab – an Eastern Cooperative Oncology Group Study. Clin Cancer Res 14(5): 1407–1412 18316562 10.1158/1078-0432.CCR-07-1154

[bib9] Duda DG, Cohen KS, di Tomaso E, Au P, Klein RJ, Scadden DT, Willett CG, Jain RK (2006) Differential CD146 expression on circulating versus tissue endothelial cells in rectal cancer patients: implications for circulating endothelial and progenitor cells as biomarkers for antiangiogenic therapy. J Clin Oncol 24(9): 1449–1453 16549839 10.1200/JCO.2005.04.2861 PMC2718681

[bib10] Ebos JM, Lee CR, Christensen JG, Mutsaers AJ, Kerbel RS (2007) Multiple circulating proangiogenic factors induced by sunitinib malate are tumor-independent and correlate with antitumor efficacy. Proc Natl Acad Sci USA 104(43): 17069–17074 17942672 10.1073/pnas.0708148104 PMC2040401

[bib11] Faivre S, Demetri G, Sargent W, Raymond E (2007) Molecular basis for sunitinib efficacy and future clinical development. Nat Rev Drug Discov 6(9): 734–745 17690708 10.1038/nrd2380

[bib12] Gabbianelli M, Pelosi E, Montesoro E, Valtieri M, Luchetti L, Samoggia P, Vitelli L, Barberi T, Testa U, Lyman S (1995) Multi-level effects of flt3 ligand on human hematopoiesis: expansion of putative stem cells and proliferation of granulomonocytic progenitors/monocytic precursors. Blood 86(5): 1661–1670 7544638

[bib13] Gao D, Nolan D, McDonnell K, Vahdat L, Benezra R, Altorki N, Mittal V (2009) Bone marrow-derived endothelial progenitor cells contribute to the angiogenic switch in tumor growth and metastatic progression. Biochim Biophys Acta 1796(1): 33–40 19460418 10.1016/j.bbcan.2009.05.001 PMC3649840

[bib14] Gatzemeier U, Blumenschein G, Fosella F (2006) Phase II trial of single agent sorafenib in patients with advanced non-small cell lung carcinoma. J Clin Oncol 24, abstr 7002 10.1200/JCO.2009.22.0541 19652055

[bib15] Groen HJ, Smit EF, Dingemans A (2007) A phase II study of erlotinib (E) and bevacizumab (B) in patients (pts) with previously untreated stage IIIB/IV non-small cell lung cancer (NSCLC). J Clin Oncol (Meeting Abstracts) 25(18_suppl): 7625

[bib16] Herbst RS, O’Neill VJ, Fehrenbacher L, Belani CP, Bonomi PD, Hart L, Melnyk O, Ramies D, Lin M, Sandler A (2007) Phase II study of efficacy and safety of bevacizumab in combination with chemotherapy or erlotinib compared with chemotherapy alone for treatment of recurrent or refractory non small-cell lung cancer. J Clin Oncol 25(30): 4743–4750 17909199 10.1200/JCO.2007.12.3026

[bib17] Hirschi KK, Ingram DA, Yoder MC (2008) Assessing identity, phenotype, and fate of endothelial progenitor cells. Arterioscler Thromb Vasc Biol 28(9): 1584–1595 18669889 10.1161/ATVBAHA.107.155960 PMC5244813

[bib18] Ingram DA, Caplice NM, Yoder MC (2005) Unresolved questions, changing definitions, and novel paradigms for defining endothelial progenitor cells. Blood 106(5): 1525–1531 15905185 10.1182/blood-2005-04-1509

[bib19] Jain RK, Duda DG (2003) Role of bone marrow-derived cells in tumor angiogenesis and treatment. Cancer Cell 3(6): 515–516 12842078 10.1016/s1535-6108(03)00138-7

[bib20] Karrison TG, Maitland ML, Stadler WM, Ratain MJ (2007) Design of phase II cancer trials using a continuous endpoint of change in tumor size: application to a study of sorafenib and erlotinib in non small-cell lung cancer. J Natl Cancer Inst 99(19): 1455–1461 17895472 10.1093/jnci/djm158

[bib21] Kerbel RS (2008) Tumor angiogenesis. N Engl J Med 358(19): 2039–2049 18463380 10.1056/NEJMra0706596 PMC4542009

[bib22] Kumar R, Crouthamel MC, Rominger DH, Gontarek RR, Tummino PJ, Levin RA, King AG (2009) Myelosuppression and kinase selectivity of multikinase angiogenesis inhibitors. Br J Cancer 101(10): 1717–1723 19844230 10.1038/sj.bjc.6605366 PMC2768111

[bib23] Le Tourneau C, Vidal L, Siu LL (2008) Progress and challenges in the identification of biomarkers for EGFR and VEGFR targeting anticancer agents. Drug Resist Updat 11(3): 99–109 18515176 10.1016/j.drup.2008.04.001

[bib24] Lind JS, Dingemans AC, Groen HJ, Smit EF (2009a) A phase II study of erlotinib and sorafenib in chemotherapy-naive patients with locally advanced/metastatic non-small cell lung cancer (NSCLC). J Clin Oncol 27, abstr 8018

[bib25] Lind JS, Lagerwaard FJ, Smit EF, Senan S (2009b) Phase I study of concurrent whole brain radiotherapy and erlotinib for multiple brain metastases from non-small-cell lung cancer. Int J Radiat Oncol Biol Phys 74(5): 1391–1396 19289264 10.1016/j.ijrobp.2008.10.026

[bib26] Loupakis F, Falcone A, Masi G, Fioravanti A, Kerbel RS, Del TM, Bocci G (2007) Vascular endothelial growth factor levels in immunodepleted plasma of cancer patients as a possible pharmacodynamic marker for bevacizumab activity. J Clin Oncol 25(13): 1816–1818 17470880 10.1200/JCO.2006.10.3051

[bib27] Manegold C (2008) Bevacizumab for the treatment of advanced non-small-cell lung cancer. Expert Rev Anticancer Ther 8(5): 689–699 18471042 10.1586/14737140.8.5.689

[bib28] Mehra N, Penning M, Maas J, Beerepoot LV, van DN, van Gils CH, Giles RH, Voest EE (2006) Progenitor marker CD133 mRNA is elevated in peripheral blood of cancer patients with bone metastases. Clin Cancer Res 12(16): 4859–4866 16914572 10.1158/1078-0432.CCR-06-0422

[bib29] Norden-Zfoni A, Desai J, Manola J, Beaudry P, Force J, Maki R, Folkman J, Bello C, Baum C, DePrimo SE, Shalinsky DR, Demetri GD, Heymach JV (2007) Blood-based biomarkers of SU11248 activity and clinical outcome in patients with metastatic imatinib-resistant gastrointestinal stromal tumor. Clin Cancer Res 13(9): 2643–2650 17473195 10.1158/1078-0432.CCR-06-0919

[bib30] Pathak AP, Hochfeld WE, Goodman SL, Pepper MS (2008) Circulating and imaging markers for angiogenesis. Angiogenesis 11(4): 321–335 18925424 10.1007/s10456-008-9119-z

[bib31] Pennell NA, Lynch Jr TJ (2009) Combined inhibition of the VEGFR and EGFR signaling pathways in the treatment of NSCLC. Oncologist 14(4): 399–411 19357226 10.1634/theoncologist.2008-0276

[bib32] Peters BA, Diaz LA, Polyak K, Meszler L, Romans K, Guinan EC, Antin JH, Myerson D, Hamilton SR, Vogelstein B, Kinzler KW, Lengauer C (2005) Contribution of bone marrow-derived endothelial cells to human tumor vasculature. Nat Med 11(3): 261–262 15723071 10.1038/nm1200

[bib33] Presta LG, Chen H, O’Connor SJ, Chisholm V, Meng YG, Krummen L, Winkler M, Ferrara N (1997) Humanization of an anti-vascular endothelial growth factor monoclonal antibody for the therapy of solid tumors and other disorders. Cancer Res 57(20): 4593–4599 9377574

[bib34] Sandler A, Gray R, Perry MC, Brahmer J, Schiller JH, Dowlati A, Lilenbaum R, Johnson DH (2006) Paclitaxel-carboplatin alone or with bevacizumab for non-small-cell lung cancer. N Engl J Med 355(24): 2542–2550 17167137 10.1056/NEJMoa061884

[bib35] Schiller J, Lee J, Hanna N, Traynor A, Carbone D (2008) A randomized discontinuation phase II study of sorafenib versus placebo in patients with non-small cell lung cancer who have failed at least two prior chemotherapy regimens: E2501. J Clin Oncol 26, abstr 8014

[bib36] Shaked Y, Ciarrocchi A, Franco M, Lee CR, Man S, Cheung AM, Hicklin DJ, Chaplin D, Foster FS, Benezra R, Kerbel RS (2006) Therapy-induced acute recruitment of circulating endothelial progenitor cells to tumors. Science 313(5794): 1785–1787 16990548 10.1126/science.1127592

[bib37] Shojaei F, Ferrara N (2008) Role of the microenvironment in tumor growth and in refractoriness/resistance to anti-antiogenic therapies. Drug Resist Updat 11(6): 219–230 18948057 10.1016/j.drup.2008.09.001

[bib38] Therasse P, Arbuck SG, Eisenhauer EA, Wanders J, Kaplan RS, Rubinstein L, Verweij J, Van GM, van Oosterom AT, Christian MC, Gwyther SG (2000) New guidelines to evaluate the response to treatment in solid tumors. European Organization for Research and Treatment of Cancer, National Cancer Institute of the United States, National Cancer Institute of Canada. J Natl Cancer Inst 92(3): 205–216 10655437 10.1093/jnci/92.3.205

[bib39] Timmermans F, Plum J, Yoder MC, Ingram DA, Vandekerckhove B, Case J (2008) Endothelial progenitor cells: identity defined? J Cell Mol Med 13(1): 87–102 10.1111/j.1582-4934.2008.00598.x PMC3823038 19067770

[bib40] Timmermans F, Van Hauwermeiren F, De Smedt M, Raedt R, Plasschaert F, De Buyzere ML, Gillebert TC, Plum J, Vandekerckhove B (2007) Endothelial outgrowth cells are not derived from CD133+ cells or CD45+ hematopoietic precursors. Arterioscler Thromb Vasc Biol 27(7): 1572–1579 17495235 10.1161/ATVBAHA.107.144972

[bib41] Verweij J, Therasse P, Eisenhauer E (2009) Cancer clinical trial outcomes: any progress in tumour-size assessment? Eur J Cancer 45(2): 225–227 19068275 10.1016/j.ejca.2008.10.025

[bib42] Vroling L, van der Veldt AAM, de Haas RR, Haanen JB, Schuurhuis GJ, Kuik DJ, van Cruijsen H, Verheul HM, van den Eertwegh AJ, Hoekman K, Boven E, van Hinsbergh VWM, Broxterman HJ (2009) Increased numbers of small circulating endothelial cells in renal cell cancer patients treated with sunitinib. Angiogenesis 12(1): 69–79 19212818 10.1007/s10456-009-9133-9

[bib43] Vroling L, Yuana Y, Schuurhuis GJ, van Hinsbergh VWM, Gundy C, de Haas R, van Cruijsen H, Boven E, Hoekman K, Broxterman HJ (2007) VEGFR2 expressing circulating (progenitor) cell populations in volunteers and cancer patients. Thromb Haemost 98(2): 440–450 17721629

[bib44] Willett CG, Boucher Y, di Tomaso E, Duda DG, Munn LL, Tong RT, Chung DC, Sahani DV, Kalva SP, Kozin SV, Mino M, Cohen KS, Scadden DT, Hartford AC, Fischman AJ, Clark JW, Ryan DP, Zhu AX, Blaszkowsky LS, Chen HX, Shellito PC, Lauwers GY, Jain RK (2004) Direct evidence that the VEGF-specific antibody bevacizumab has antivascular effects in human rectal cancer. Nat Med 10(2): 145–147 14745444 10.1038/nm988 PMC2693485

[bib45] Yoder MC, Ingram DA (2009) Endothelial progenitor cell: ongoing controversy for defining these cells and their role in neoangiogenesis in the murine system. Curr Opin Hematol 16(4): 269–273 19417649 10.1097/MOH.0b013e32832bbcab

[bib46] Zhu AX, Sahani DV, Duda DG, di TE, Ancukiewicz M, Catalano OA, Sindhwani V, Blaszkowsky LS, Yoon SS, Lahdenranta J, Bhargava P, Meyerhardt J, Clark JW, Kwak EL, Hezel AF, Miksad R, Abrams TA, Enzinger PC, Fuchs CS, Ryan DP, Jain RK (2009) Efficacy, safety, and potential biomarkers of sunitinib monotherapy in advanced hepatocellular carcinoma: a phase II study. J Clin Oncol 27(18): 3027–3035 19470923 10.1200/JCO.2008.20.9908 PMC2702235

[bib47] Zurita A, Jonasch E, Wu H, Tran H, Heymach J (2009) Circulating biomarkers for vascular endothelial growth factor inhibitors in renal cell carcinoma. Cancer 115(10): 2346–2354 19402074 10.1002/cncr.24228 PMC4407476

